# Design Multilayer Antireflection Coatings for Terrestrial Solar Cells

**DOI:** 10.1155/2014/265351

**Published:** 2014-01-28

**Authors:** Feng Zhan, Zhipeng Li, Xiaoming Shen, Huan He, Jianmin Zeng

**Affiliations:** ^1^The Key Laboratory of Nonferrous Metal Materials and New Processing Technology of Ministry of Education, Guangxi University, Nanning 530004, China; ^2^Department of Physics and Astronomy, University of North Carolina, Chapel Hill, NC 27599, USA

## Abstract

In order to analyze the influence of methods to design antireflection coatings (ARCs) on reflectivity of broadband solar cells, we provide detailed analyses about the ARC coupled with a window layer and the refractive index dispersion effect of each layer. By multidimensional matrix data simulation, two methods were employed to measure the composite reflection of a SiO_2_/ZnS double-layer ARC within the spectral ranges of 300–870 nm (dual junction) and 300–1850 nm (triple junction) under AM1.5 solar radiation. A comparison study, between the results obtained from the commonly used weighted average reflectance method (WAR) and that from the introduced effective average reflectance method (EAR), shows that the optimization of ARC by EAR method is convenient and feasible.

## 1. Introduction

Reflective loss of the incident light on the device surface is an important factor that affects efficiency of solar cell (SC). Design of antireflection structure has become a key factor in SC fabrication [1–7]. For III–V tandem SCs, a proper group of transparent materials are deposited on the SC surface, leading to the formation of antireflection coatings (ARCs) [7, 8]. Consequently, the reflective loss of the incident lights would be minimized and the SCs work in a spectral range with higher efficiency. Therefore, broadband ARCs are essential for SCs operating at a broad spectrum.

Generally, there are various types of broadband ARC. For instance, flat multilayer ARCs can be double, triple, or quadruple layer. Many studies have shown that the performance of a single layer coating is not satisfying due to its narrow working spectral range. Double-, triple-, or even multiple-layer ARCs [7, 9–11] have better performance when referring to broadband SCs. Double-layer ARCs are commonly used because of their simple fabrication process and low cost. In this paper, the principle and method of antireflective films were introduced. A multidimensional matrix for the refractive index dispersion effect of each layer was used to simulate the reflectivity of several optical film systems. An effective average reflectance *R*_*e*_ method (EAR) simplified from the commonly used weighted average reflectance *R*_*w*_ method (WAR) was also utilized to design ARCs. The optimizations of ARCs in the dual- and triple-junction SCs by two methods were compared and analyzed. Accordingly, it demonstrates that optimizing ARC by minimizing *R*_*e*_ is more convenient under the AM1.5 conditions. The models for optimization of multilayer ARC are presented in the following section, and their differences from a typical double-layer ARC are discussed in detail thereafter.

## 2. Methodology

The reflectivity at normal incidence of a SiO_2_/ZnS double-layer antireflection coating (ARC) on an Al_0.5_In_0.5_P window layer is typically modeled in front of a Ga_0.5_In_0.5_P top cell. This approach is based on previously measured optical parameters for materials such as Al_0.5_In_0.5_P and Ga_0.5_In_0.5_P [12], as well as others [12–14] used as ARCs. The optical parameters of any composition of ARC are generally determined by cubic interpolation. We adopted the transfer-matrix method to model the reflectance of the system, as this approach can be used to analyze multilayer films of varying thickness, refractive indices (*n*), and extinction coefficients (*k*) on a substrate.

**Figure 1:**
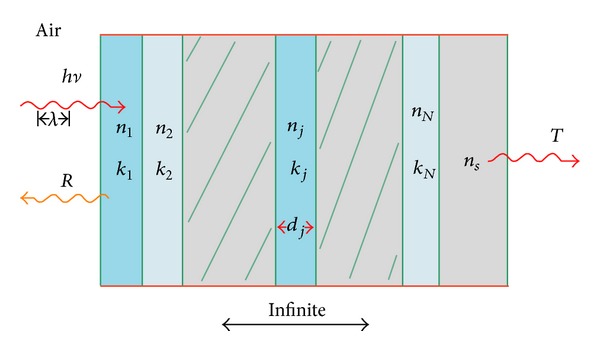
Schematic diagram of the optical model for stacked films.

Optical interference matrix is an effective way to calculate reflectivity of film. To allow the situation that the incident angle of light is zero degree, considering the optical thin film system of *N* layers prepared on the substrate as shown in Figure 1, *n*_*j*_ is the refractive index and *k*_*j*_ is the extinction coefficient, *d*_*j*_ is the thickness in each layer, respectively, and *n*_0_ is refractive index of air (*n*_0_ = 1). According to the refractive index and thickness of each layer, interference matrix of each layer can be determined. A characteristic matrix formulation of the film system is obtained by multiplying interference matrix of each layer [15]:

(1) [BC]={∏j=1N[cosδjisinδjnjinjsinδjcosδj]}[1ns]=[M11M12M21M22][1ns],

where *δ*_*j*_ is the effective optical thickness of the layer at a given wavelength. The 2*δ*_*j*_ is equal to the phase difference of two adjacent coherent light beams. *Y* = *C*/*B* is the optical admittance. The reflectivity *R* of the whole film system is expressed as

(2) $$\begin{array}{c} R={\left|r\right|}^{2}={\left|\frac{n_{0}-Y}{n_{0}+Y}\right|}^{2}. \end{array}$$

The solar spectrum spans a broad range of wavelengths. To enable more incident light to enter the SC, the internal quantum efficiency of the material and the sun's spectral characteristics should all be considered during the design of ARCs. The weighted average reflectivity within the entire spectrum can be calculated by the incident photon flux *F*(*λ*) [16], the internal quantum efficiency (IQE) of the SC *Q*(*λ*) [17, 18], and the reflectivity of monochromatic light *R*(*λ*) [19]:

(3) $$\begin{array}{c} R_{w}=\frac{\int_{\lambda_{1}}^{\lambda_{2}}F\left(\lambda \right)R\left(\lambda \right)Q\left(\lambda \right)d\lambda }{\int_{\lambda_{1}}^{\lambda_{2}}F\left(\lambda \right)Q\left(\lambda \right)d\lambda }, \end{array}$$

where *λ*_1_ represents the lower limit of spectral response and *λ*_2_ represents the upper limit.

Considering that there are minor differences in IQEs among different monochromatic light in practical applications, we set IQEs equal to induce the average effective reflectance *R*_*e*_ for a convenient design [20]:

(4) $$\begin{array}{c} R_{e}=\frac{\int_{\lambda_{1}}^{\lambda_{2}}F\left(\lambda \right)R\left(\lambda \right)d\lambda }{\int_{\lambda_{1}}^{\lambda_{2}}F\left(\lambda \right)d\lambda }. \end{array}$$

## 3. Results and Discussion

In this section, we will discuss the minimization results obtained for the weighted average reflectance and effective average reflectance of the SiO_2_/ZnS double-layer structure on double- and triple-junction SCs under AM1.5 conditions.

### 3.1. Dual Junction Solar Cell (300–870 nm)

**Figure 2:**
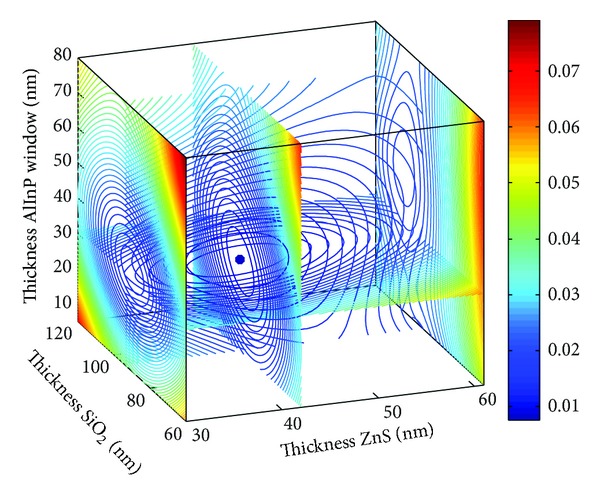
SiO_2_/ZnS ARC *R*_*e*_ versus films thickness.

**Figure 3:**
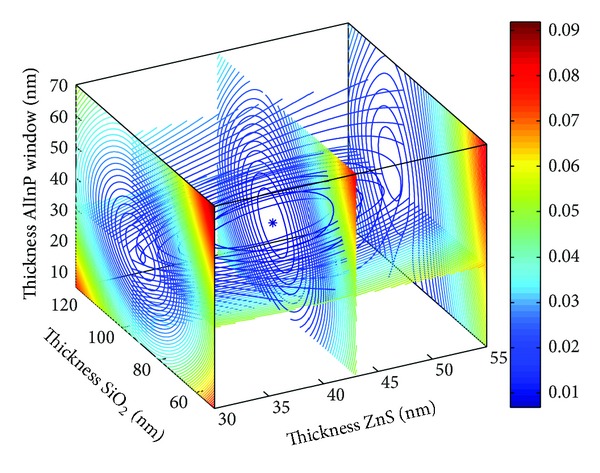
SiO_2_/ZnS ARC *R*_*w*_ versus films thickness.

**Figure 4:**
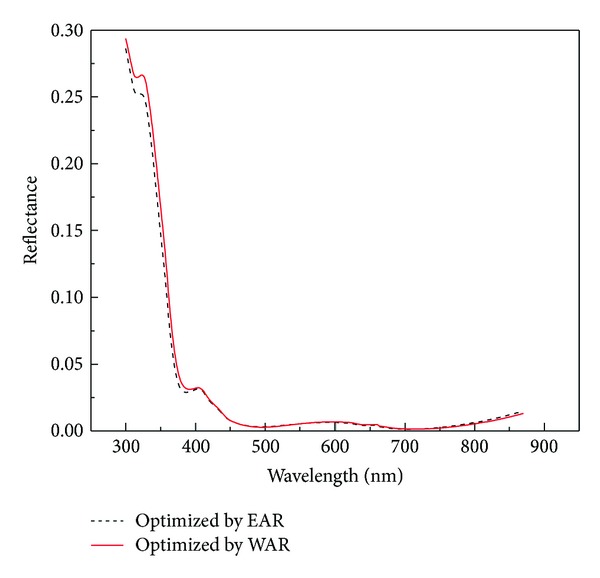
Optimal SiO_2_/ZnS ARC reflectivity versus wavelength.

The four-dimensional images shown in Figures 2 and 3 depict the optimal parameters of the SiO_2_/ZnS ARC for the Ga_0.5_In_0.5_P/GaAs double-junction SC under AM1.5 conditions. The reflectivity curves of SiO_2_/ZnS films optimized by *R*_*e*_ and *R*_*w*_ are shown in Figure 4. The SiO_2_/ZnS ARC parameters for the double-junction SC optimized by different methods are summarized in Table 1.

Those optimized by *R*_*e*_ and *R*_*w*_ exhibited minimal differences (see in Table 1), with the change in thickness values being less than 2 nm and the reflectivities in the spectral range of 400–700 nm remaining almost unchanged, that these reflectivities are the lowest where the solar photon flux is mainly distributed (see Figure 4). These results indicated that optimizing the ARC by minimizing the effective average reflectance *R*_*e*_ for double-junction SC is feasible.

### 3.2. Triple Junction Solar Cell (300–1850 nm)

**Figure 5:**
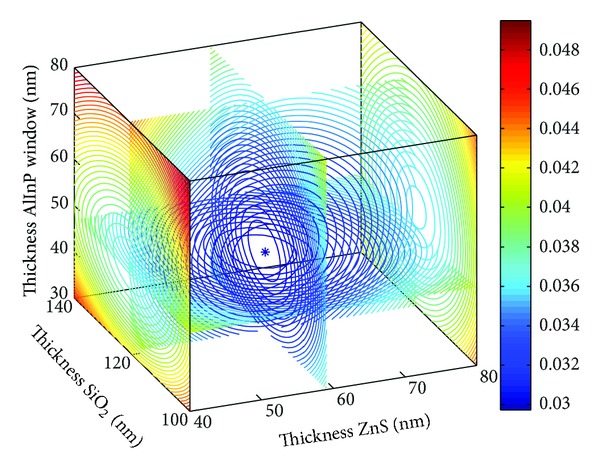
SiO_2_/ZnS ARC *R*_*e*_ versus films thickness.

**Figure 6:**
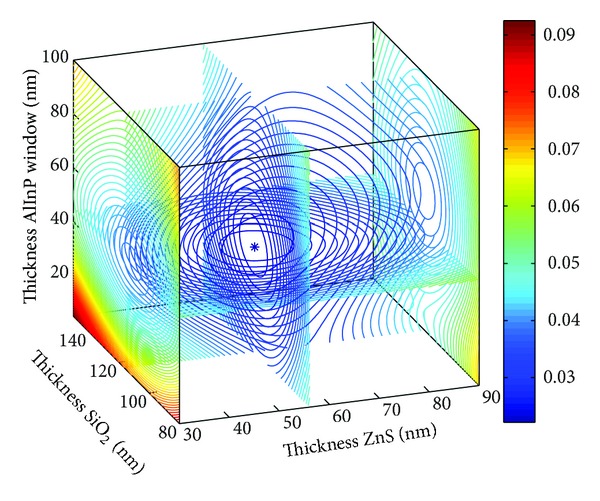
SiO_2_/ZnS ARC *R*_*w*_ versus films thickness.

**Figure 7:**
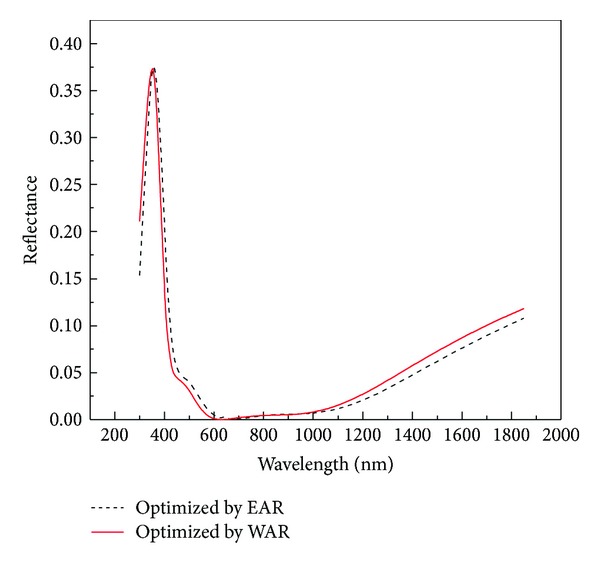
Optimal SiO_2_/ZnS ARC reflectivity versus wavelength.

The four-dimensional images shown in Figures 5 and 6 depict the optimal parameters of the SiO_2_/ZnS ARC for the Ga_0.5_In_0.5_P/GaAs/Ge triple-junction SC under AM1.5 conditions. The reflectivity curves of SiO_2_/ZnS films optimized by *R*_*e*_ and *R*_*w*_ are shown in Figure 7. The SiO_2_/ZnS ARC parameters for the triple-junction SC optimized by different methods are summarized in Table 2.

Those optimized by WAR and EAR methods also exhibited minimal differences in triple junction SC (see in Table 2), with the change in thickness values being less than 2 nm and the reflectivities in the spectral range of 400–700 nm remaining almost unchanged. The reflectivities are properly the lowest where the solar photon flux is mainly distributed (see Figure 7). These results indicated that optimizing the ARC by minimizing the effective average reflectance *R*_*e*_ for double-junction SC is feasible.

## 4. Summary

To summarize, this study represents the theoretical optimization of the SiO_2_/ZnS double-layer ARC on the Al_0.5_In_0.5_P window layer for double- and triple-junction SCs under AM1.5 condition. It demonstrated that there are no considerable differences in the final optimal ARC parameters by WAR method and EAR method. For the double-junction SC, the changes in ARC thickness and average reflectivity were determined to be less than 2 nm and 0.07%, respectively. On the other hand, for the triple-junction SC, the corresponding values obtained were less than 6 nm and 0.76%, respectively. These slight changes in the parameters are acceptable. Therefore, optimizing ARCs by minimizing the *R*_*e*_ introduced, instead of *R*_*w*_, as is commonly used, is thus a viable technique under AM1.5 condition.

## Figures and Tables

**Table 1:** Comparison of parameters of SiO_2_/ZnS ARC for dual junction SC optimized by different methods.

	Optimized by EAR method	Optimized by WAR method	Change
SiO_2_ thickness (nm)	94	95	−1.05%
ZnS thickness (nm)	42	43	−2.32%
Window layer thickness (nm)	31	31	0%

Average reflectance (*R*_*e*_/*R*_*w*_)	0.75%	0.68%	0.07%

**Table 2:** Comparison of parameters of SiO_2_/ZnS ARC for triple junction SC optimized by different methods.

	Optimized by EAR method	Optimized by WAR method	Change
SiO_2_ thickness (nm)	121	116	4.31%
ZnS thickness (nm)	59	56	5.36%
Window layer thickness (nm)	47	44	6.82%

Average reflectance (*R*_*e*_/*R*_*w*_)	2.97%	2.21%	0.76%
